# The Role of Zinc on Liver Fibrosis by Modulating ZIP14 Expression Throughout Epigenetic Regulatory Mechanisms

**DOI:** 10.1007/s12011-023-04057-5

**Published:** 2024-01-15

**Authors:** Zeynep Busra Aksoy-Ozer, Ceylan Verda Bitirim, Belma Turan, Kamil Can Akcali

**Affiliations:** 1 Ankara University Stem Cell Institute, Ankara, Turkey; 2 Biophysics Department, Lokman Hekim University Medical School, Ankara, Turkey; 3 Biophysics Department, Ankara University Medical School, Ankara, Turkey

**Keywords:** Liver fibrosis, Zinc, Epigenetics, HDAC, Acetylation

## Abstract

Zinc plays a pivotal role in tissue regeneration and maintenance being as a central cofactor in a plethora of enzymatic activities. Hypozincemia is commonly seen with chronic liver disease and is associated with an increased risk of liver fibrosis development and hepatocellular carcinoma. Previously favorable effects of zinc supplementation on liver fibrosis have been shown. However, the underlying mechanism of this effect is not elucidated. Liver fibrosis was induced in mice by using CCl_4_ injection, followed by treatment with zinc chloride (ZnCl_2_) both at fibrotic and sham groups, and their hepatocytes were isolated. Our results showed that the administration of ZnCl_2_ restored the depleted cytosolic zinc levels in the hepatocytes isolated from the fibrotic group. Also, alpha-smooth muscle actin (*αSMA*) expression in hepatocytes was decreased, indicating a reversal of the fibrotic process. Notably, ZIP14 expression significantly increased in the fibrotic group following ZnCl_2_ treatment, whereas in the sham group ZIP14 expression decreased. Chromatin immunoprecipitation (ChIP) experiments revealed an increased binding percentage of Metal-regulatory transcription factor 1 (MTF1) on *ZIP14* promoter in the hepatocytes isolated from fibrotic mice compared to the sham group after ZnCl_2_ administration. In the same group, the binding percentage of the histone deacetylase HDAC4 on *ZIP14* promoter decreased. Our results suggest that the ZnCl_2_ treatment ameliorates liver fibrosis by elevating intracellular zinc levels through MTF1-mediated regulation of *ZIP14* expression and the reduction of *ZIP14* deacetylation via HDAC4. The restoration of intracellular zinc concentrations and the modulation of *ZIP14* expression by zinc orchestrated through MTF1 and HDAC4, appear to be essential determinants of the therapeutic response in hepatic fibrosis. These findings pave the way for potential novel interventions targeting zinc-related pathways for the treatment of liver fibrosis and associated conditions.

## Introduction

Liver fibrosis represents the initial stage of hepatocellular carcinoma (HCC) and often remains clinically asymptomatic, posing challenges for early detection and intervention [1]. Following hepatic injury, activated hepatic satellite cells (HSCs) transform into myofibroblast-like cells, which play a central role in the accumulation of extracellular matrix (ECM) proteins and the formation of scar tissue [2, 3]. Hepatic fibrosis and subsequent HCC are associated with reduced serum zinc levels. Both in vivo and in vitro studies have provided evidence of the beneficial impact of zinc supplementation on liver fibrosis [4]. Nevertheless, the precise molecular mechanisms underlying the anti-fibrotic effects of zinc treatment have yet to be fully elucidated. Therefore, a comprehensive investigation into the differential responses of fibrotic cells to zinc administration is warranted to gain deeper insights into the cellular healing mechanisms mediated by zinc in the context of liver fibrosis.

The pivotal role of zinc in maintaining robust physiological continuity is widely acknowledged. Bioinformatic investigations have elucidated that a notable fraction, approximately 10%, of the protein repertoire encoded within the human genome exhibits the capacity to engage with zinc ions [5, 6]. Zinc has a critical regulatory function in orchestrating enzymatic and transcriptional activities essential to cellular processes [7–10]. Effective zinc binding necessitates the presence of specific structural motifs, with zinc finger domains constituting foremost examples. Intracellular zinc homeostasis is predominantly regulated through zinc transporter proteins [11]. Investigations have revealed associations between impaired zinc transporters and certain clinical disorders [12]. Consequently, pharmaceutical agents targeting these transporter proteins located within cellular or organelle membranes are believed to hold substantial therapeutic potential [13, 14]. In the human body, zinc is found in quantities of approximately 2–3 g, contingent upon body dimensions. Approximately 5% of stored zinc is localized within the hepatic reservoir. The zinc content in blood serum is less than 1%, with approximately 80% of serum zinc bound to albumin. The body is capable of regulating an amount of zinc approximately 10 times the daily recommended intake [14]. Variations in zinc content between tissues or cell types can be attributed to distinct zinc transporter protein types and quantities inherent to different cell types. The maintenance of the homeostasis of intracellular free (labile) zinc ion levels ([Zn^2+^]_i_) relies on the activity of zinc transporter proteins located on the cell membrane and within organelles. Although the total intracellular zinc concentration may reach levels of 10–100 µM, the pool of free zinc within the cytoplasm is often present at pico-molar, and at times, nano-molar levels. This phenomenon predominantly stems from the propensity of zinc to bind to other proteins within the cellular milieu or to be sequestered within organelles [16–18].

Zinc ions exist in a stable divalent cationic form within living organisms, thereby obviating the requirement for redox reactions to facilitate their translocation across cellular membranes [18]. In humans, 14 zinc regulated transporter/iron regulated transporter-like protein (Zrt/Irt-) like protein (ZIP) and 10 zinc transporter (ZnT) channel proteins have been identified, with ZIPs facilitating the transfer of Zn^2+^ from intracellular stores or the extracellular environment to the cytoplasm, while ZnTs mediate the efflux of Zn^2+^ from the cytoplasm or sequester them into organelles. The pivotal significance of zinc in physiological and cellular processes necessitates the tight regulation of ZnT and ZIP transporter protein expressions. Transcriptional and post-transcriptional regulations respond to stimuli such as hormones, cytokines, endoplasmic reticulum (ER) stress, oxidative stress, and hypoxia [22–25]. As an illustrative example, a study carried out in the year 2017 delineated the causative relationship between ER stress and heightened expression of ZIP14. This increased expression was found to augment hepatic zinc accumulation. Intriguingly, the study further unveiled that the facilitation of zinc transport through the agency of ZIP14 serves as a mitigating factor against apoptosis and steatosis development [19]. In order to identify the channel proteins responsible for zinc homeostasis during acute inflammation in the liver, the mRNA expressions of ZIP and ZnT channels were measured in mice where liver inflammation was induced by injecting LPS. The mRNA expression of *ZIP14* was observed to increase by 3.1-fold [20].

Intracellular zinc homeostasis also involves the orchestrated interplay of organelles and proteins known as metallothioneins (MTs). MTs are small proteins and have cysteine-rich content, exhibit an elevated affinity for sequestering zinc ions. In addition to their roles in mitochondria and the ER, MTs serve as major intracellular zinc reservoirs [21]. As the amount of intracellular metal ions increases, MT proteins release the bound zinc ions, thereby raising the intracellular free zinc concentration. The elevated zinc concentration activates MTF-1, which plays a crucial role in balancing cellular metal ion levels. MTF-1 recognizes genes with a metal-responsive element (MRE: TGCRCNC) sequence in their promoter regions and binds to them, thereby facilitating their transcriptional regulation [27, 28]. MT proteins are among the proteins regulated by MTF-1 expression. It is known that conditions such as oxidative stress and hypoxia also enhance MTF-1 activity [30–32]. Importantly, MTF-1’s binding to the MRE sequence is contingent on the availability of sufficient zinc concentration [26]. In a study conducted in 2015, it was demonstrated that MTF-1 plays a role in increasing ECM deposition and enhances fibrosis formation [27].

Zinc plays a role in the activation of many proteins involved in epigenetic regulations such as DNA methylation and histone modifications. It is known that many epigenetic regulators, including DNA methyltransferases (DNMTs), histone demethylases, histone acetyltransferases (HATs), and histone deacetylases (HDACs) contain zinc finger domains [34, 35]. HDACs are responsible for the deacetylation of histones. Histone acetylation is one of the modifications that activate gene expression [30]. HDAC4 is a class IIa HDAC and it is known that HDAC4 plays an important role in ECM remodeling and the regulation of ECM deposition in fibrosis [31].

Since zinc plays an important role for the activity of many proteins including transcription factors, we hypothesized that zinc shows its effect through genetic and epigenetic modulations involving the ZIP14 channel protein. This study provides compelling evidence that zinc regulates the interaction between MTF-1 and ZIP14. Also, zinc shows epigenetic modulatory role by interacting with HDAC4 and influencing the activity of HDAC4 on *ZIP14* promoter during liver fibrosis. The primary discovery of this research is that the introduction of zinc supplementation to mice with liver fibrosis shows a promising response, potentially reversing fibrotic characteristics by influencing the ZIP14 channel protein. This modulation is observed at both genetic and epigenetic levels. The maintenance of intracellular zinc homeostasis ([Zn2+]i) is predominantly achieved through zinc transporters situated on cell membranes and organelle membranes. Our investigation confirmed the existence of metal-responsive element (MRE) sequences on the *ZIP14* gene promoter, and notably, our results indicate that MTF-1 regulates ZIP14 when zinc supplementation is applied to the fibrosis groups. This finding implies that MTF-1 may function as a genetic regulator of zinc’s therapeutic impact on fibrosis. Furthermore, we have also identified indications that ZIP14 expression undergoes epigenetic regulation in hepatic fibrosis. The findings of this study may shed new light on the molecular pathways through which zinc therapy exerts its beneficial effects on liver fibrosis and bring potential avenues for novel interventions targeting zinc-related signaling pathways in the treatment of liver fibrosis and its related conditions.

## Materials and Methods

### Mouse Model

All procedures were reviewed and approved by the Institutional Animal Care and Use Committee of Ankara University and complied with the Guide for the Use and Care of Laboratory Animals published by the National Institutes of Health. 6-week-old male c57bl/6 mice were used to induce a liver fibrosis model by using 10% CCl_4_ injection subcutaneously for 8 weeks. Sham groups were injected with the same amount of mineral oil (Merck, #5904). ZnCl_2_ treated groups were injected with ZnCl_2_ (Merck, #7646-85-7). ZnCl_2_ was diluted in mineral oil at 10 µM concentration, and treatment was applied for two weeks by subcutaneous injection on both control and fibrosis groups. At the end of 10 weeks of treatment (8 weeks for CCl_4_ injection and 2 weeks for ZnCl_2_ injection), all groups were anesthetized with sodium pentobarbital, and hepatocyte isolation was done by whole-body perfusion.

### Hepatocyte Isolation

After opening the abdomen of the mice anesthetized with sodium pentobarbital, we entered the heart with a 19-gauge syringe needle from the left ventricle. Animals were perfused with EGTA solution (4 mM, pH 8.2 in Hank’s Balanced Salt Solution, HBSS) through the left ventricle. After the perfusion, the digestion medium (15 mM HEPES, 0.8 mg/mL Collagenase Type IV (Gibco, #17,104,019) in Dulbecco’s Modified Eagle’s Medium (DMEM)) was given in the same way and the liver cells were released into the tissue without disrupting the integrity of the liver. The gallbladder was removed without rupture and the liver was dissected. The liver was taken into a sterile petri dish and washed with isolation solution (10% FBS, 1% pen/strep, in 1X Dulbecco’s Phosphate Buffer Saline (DPBS)) and mechanically dissected with tweezers. The released cells were collected in an isolation medium and transferred to a 50 mL canonical tube, washed three times at 50xg for 1 min at 4 C. Cells counted after washing were cultured in Collagen type I (Gibco, A1064401) coated tissue flasks in culturing medium (10% FBS, 1% pen/strep, in DMEM).

### Measurement of Intracellular Labile [Zn^2+^]_i_ by Flow Cytometry

Short-lived, brief, and localized light emissions (‘sparks’) were recorded from individual cells after 40 min incubation with 1 µM FluoZin-3, AM (Thermo Scientific, F24195) respectively. Stained cells were excited at either 494 nm, and emission was collected at either 516 nm. Fluorescence intensity and change were recorded by flow cytometry (NovoCyte, California, USA). Readings were obtained with HEPES-buffered solution supplemented with zinc ionophore pyrithione (10 µM, ZnPT) to record the highest fluorescent intensity (F_max_), with the subsequent addition of 50 µM *N*, *N*, *N*′, *N*′-tetrakis (2-pyridyl methyl) ethylenediamine (50 µM, TPEN) to bring the [Zn^2+^]_i_ to zero (F_min_). After the steady-state fluorescence intensity (F) was measured, F_max_ and F_min_ fluorescence ratios related to [Zn^2+^]_i_ were also measured, and fold changes of [Zn^2+^]_i_ were calculated according to the manufacturer’s formula. The Kd value of Fluozin-3 dye is 15 nM [32]. The calculation was performed as given below:

$$\text[Z\text{n}^{2+}{]}_{\rm i}=\text{Kd } [\text ({F}-\text{F}_{\rm min})/(\text{F}_{\rm max}-\text{F})]$$

### The mRNA Analysis and Quantitative PCR

Total RNA was extracted from the hepatocytes using Riboex reagent and cDNA was synthesized using the iScript cDNA synthesis kit (Bio Rad, #1,708,890) according to manufacturer instructions. Primers for qRT-PCR were designed by using Primer Blast NCBI (Table 1). The mRNA expression levels were measured in duplicate using a 2X GoTaq Master mix (Promega, USA) based on polymerase chain reactions (PCR). The dCT was calculated relative to the average Ct value for *GAPDH* expression. The average dCT for the sham group was used as the normalization value to calculate ddCT. The ddCT was then calculated for relative fold difference in transcription levels, and relative quantification (RQ) values were calculated as 2^− ddCt^.

**Table 1:** Primers used in mouse RT-PCR experiments

Gene Name	Forward Primer (5’-3’)	Reverse Primer (5’-3’)
*ZIP14*	CCTTACCCTGACACAGCTGA	GAGATCGCTCGCTCAAGTTG
*MTF-1*	AGAGGCTTCACACAGGGAAA	TCCGGACGTGAGTTTTAAGG
*MT1A*	GCTGTCCTCTAAGCGTCACC	CCAAGGTGTCCCAACTCACT
*MT2*	CCATATCCCTTGAGCCAGAA	ATCGACGAGAGATCGGTTTG
*αSMA*	CTGACAGAGGCACCACTGAA	AGAGGCATAGAGGGACAGCA
*ALB*	ATGAAGTGGGTAACCTTTCTC	GGATGTCTTCTGGCAACTTC
*GAPDH*	ACCACAGTCCATGCCATCAC	TCCACCACCCTGTTGCTGTA

### Chromatin Immunoprecipitation (ChIP) Assay

Simple ChIP Enzymatic Chromatin IP Kit (Magnetic Beads) (Cell Signaling Technology, #9003) was used for ChIP assay according to the manufacturer’s protocol. A 5 µg of chromatin DNA was immunoprecipitated using an antibody against HDAC4 (LSbio, LS-C330857) and MTF-1 (Novus Biologicals, NBP1-86380) and 10% of DNA was used as input. ChIP assay was analyzed with quantitative PCR using qPCR 2X GoTaq Master mix (Promega, A6001) and results were expressed using the percent input method:

$$\begin{aligned} {\text{Percent input}} & = ({\text{Primer Efficiency}})^{({\text{CT input}} - {\text{CT IP sample}})} \\ & \quad \times \%\, {\text{of input}}\end{aligned}$$

### Flow Cytometry

Hepatocytes were pelleted by centrifugation at 100x g for 1 min. After this step, the cells were kept on ice and the solutions were used at + 4°C. It was dissolved in an ice-cold staining solution (150 µL of 1X DPBS containing 5% BSA and 0.05% sodium azide). A 50 µL containing 5 × 10^4^ cells as the unstained cell group was separated into a separate microcentrifuge tube and the remaining cells were stained with 1 µL of FITC mouse anti-human ZIP14 (MyBioSource, MBS151580) antibody and isotype antibodies respectively to the cells in 100 µL of 5% BSA and 0.05% sodium azide in 1X PBS. Cells were incubated for 30 min at + 4°C in the dark. Then, 1 mL of 1X DPBS was added to the stained cells and 100xg was centrifuged for 1 min, and the pellet was dissolved with 200 µL of 1X DPBS and the reading was taken in flow cytometry NovoCyte, California, USA).

### Immunofluorescence Staining

The Anti-Slc30a14 antibody (MyBioSource, MBS151580) was utilized for immunofluorescence staining of hepatocytes. Freshly isolated hepatocytes were seeded on sterile coverslips. Once the cells attached to the coverslips, the cell media was removed, and the cells were incubated with a fixative solution for 15 min at room temperature without washing. Following the removal of the fixative solution, the permeabilized solution was added and kept at room temperature for 15 min. After the permeabilized solution was removed, a blocking solution (5% BSA-TritonX, BSA-T) was added and incubated at room temperature for 1 h. The primary antibody was diluted in the blocking solution (1:200), and overnight incubation was performed at + 4 °C. The primary antibody wash was conducted three times with PBS-TritonX (PBS-T) solution. The Anti-rabbit IgG-FITC secondary antibody (MyBioSource, MBS539169) was also diluted in BSA-T (1:350) and incubated at room temperature for 1 h. After the secondary antibody was washed three times with PBS-T, coverslips were mounted on slides with mounting medium with DAPI (Abcam, ab104139). Images were captured using a Zeiss LSM-880 confocal laser scanning microscope.

### Statistical Analysis

The statistical significance between the groups of qRT-PCR was determined by the paired Student’s t-test. ChIP and flow cytometry experiments were determined by the unpaired Student’s t-test. The statistical differences between means of more than two groups were determined by the one-way ANOVA multiple comparison. Prism 8.0 software was used for all statistical analysis. The values from all experiments were expressed as the means ± SD (n = 4), and p < 0.05 was chosen as statistically significant.

## Results

**Fig. 1:**
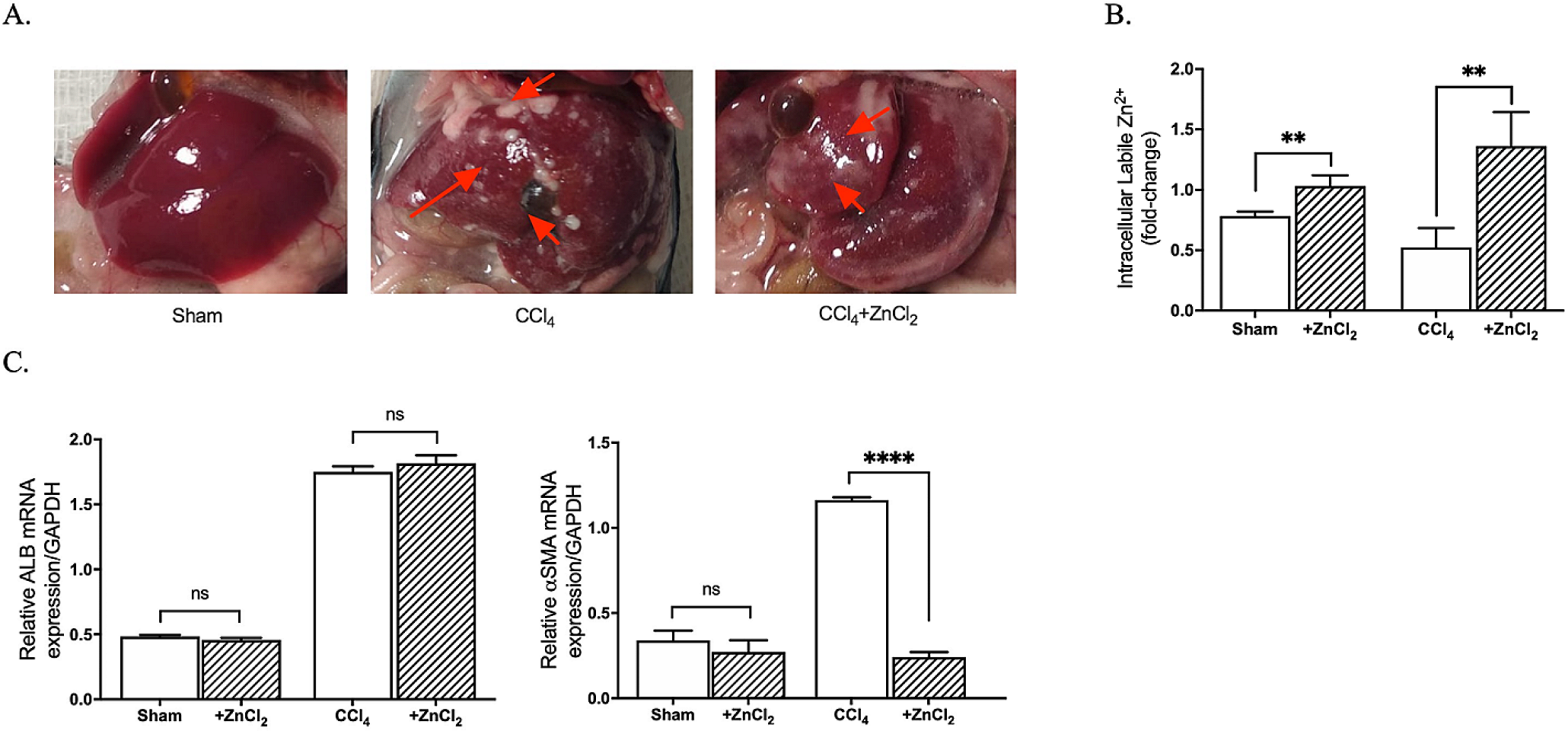
Macroscopic examination of CCl_4_-induced hepatic fibrosis, its effects on intracellular zinc levels ([Zn^2+^]_i_), and molecular confirmation of fibrosis formation **A** Liver photos were taken right before the perfusion. The Sham group received mineral oil only, CCl_4_ and CCl_4_ + ZnCl_2_ group were injected with 10% CCl_4_ in mineral oil for 8 weeks. 10 µM ZnCl_2_ was only injected to CCl_4_ + ZnCl_2_ group for two weeks after CCl_4_ treatment was completed. Red arrows show the scar tissue nodes. **B** The [Zn^2+^]_i_ of all groups were measured with Fluozin-3AM. The change in intracellular free (labile) Zn^2+^ levels after 10 µM ZnCl_2_ treatment. **C** Effects of 10 µM ZnCl_2_ injection to CCl_4_-induced hepatic fibrosis model mice on Albumin and aSMA expressions. The mRNA expression of Alb and αSMA genes are analyzed by qRT-PCR. Data are expressed as the mean ± SD (n = 4). *p < 0.05, **p < 0.01, n.s.: not significant

In this study, we established a liver fibrosis model in C57BL/6 mice through an 8-week CCl_4_ injection protocol. Macroscopic examinations conducted before hepatocyte isolation revealed the presence of fibrotic tissues resulting from CCl_4_ administration (Fig. 1A). To validate the development of liver fibrosis, we assessed the expression of alpha-smooth muscle actin (aSMA), a well-established fibrosis marker, and monitored intracellular [Zn^2+^] levels. As expected, liver fibrosis was confirmed by a significant increase in *aSMA* expression and a corresponding decrease in [Zn^2+^]_i_. Remarkably, upon supplementation with ZnCl_2_, we observed a notable reduction in *αSMA* expression in the fibrosis group, indicating a potential reversal of the fibrotic process (Fig. 1B). Conversely, the control group did not exhibit a significant change in *αSMA* expression after ZnCl_2_ treatment, suggesting a specific effect on fibrotic conditions. Additionally, we concurrently examined the expression of albumin in hepatocytes and found that ZnCl_2_ treatment did not exert a significant impact on albumin expression (Fig. 1C), thereby indicating its selective influence on fibrosis-related processes. These results underscore the potential therapeutic role of ZnCl_2_ supplementation in mitigating liver fibrosis and point towards its specific involvement in fibrosis-related signaling pathways while sparing other essential hepatocyte functions, such as albumin expression.

**Fig. 2:**
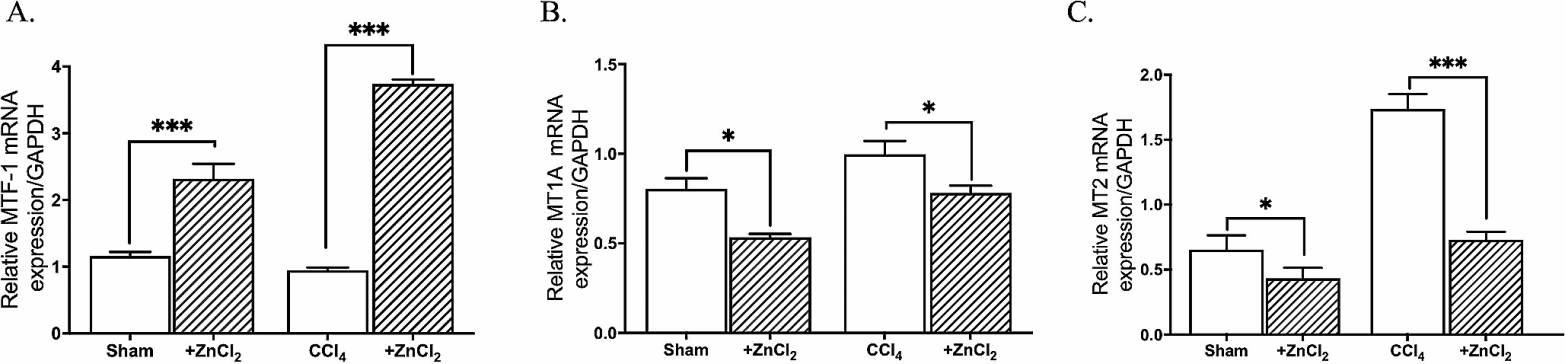
Effects of 10 µM ZnCl_2_ injection to CCl_4_-induced hepatic fibrosis model mice on intracellular zinc homeostasis regulatory genes: Sham group received mineral oil only, CCl_4_ and CCl_4_ + ZnCl_2_ group were injected with 10% CCl_4_ in mineral oil for 8 weeks. 10 µM ZnCl_2_ was only injected to CCl_4_ + ZnCl_2_ group for two weeks after CCl_4_ treatment was completed. **A** MTF-1, **B** MT1A, and **C** MT2 Data are expressed as the mean ± SD (n = 4). *p < 0.05, **p < 0.01, n.s.: not significant

**Fig. 3:**
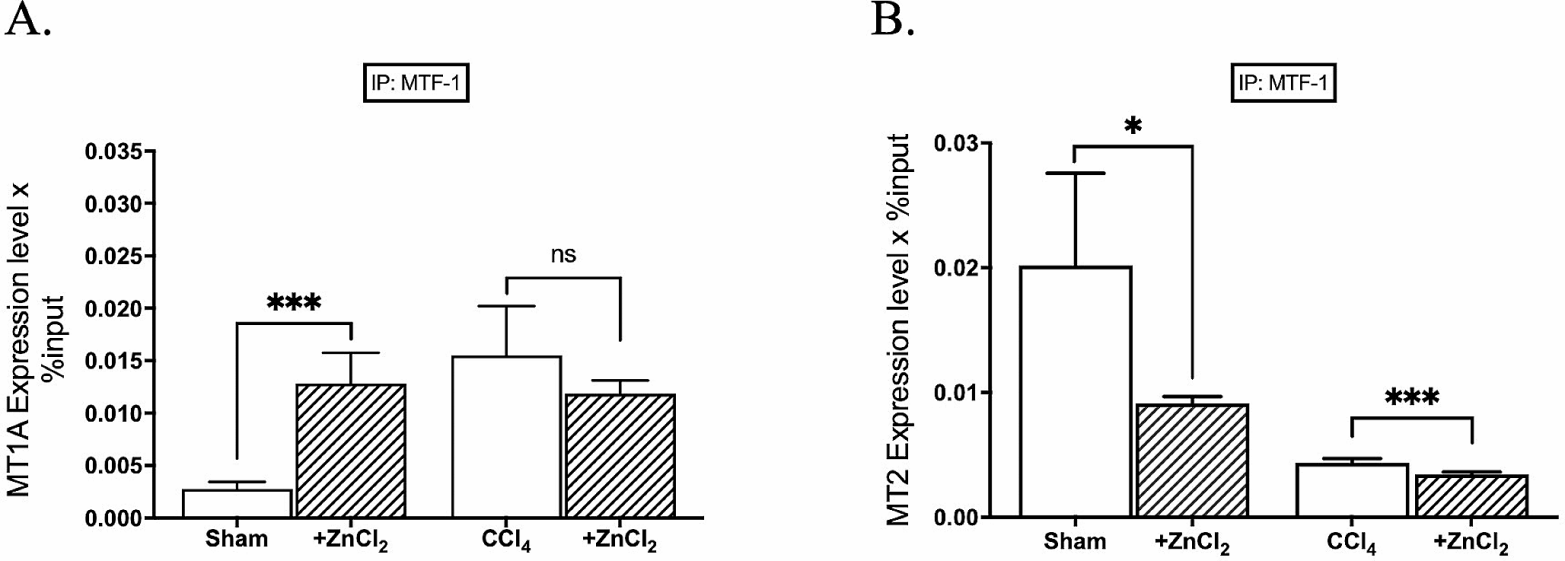
Effects of ZnCl_2_ on the accumulation of MTF-1 to the promoters of metallothionein genes. The Sham group received mineral oil only, CCl_4_ and CCl_4_ + ZnCl_2_ group were injected with 10% CCl_4_ in mineral oil for 8 weeks. A 10 µM ZnCl_2_ was only injected into the CCl_4_ + ZnCl_2_ group for two weeks after CCl_4_ treatment was completed. The enrichment of the MTF-1 transcription factor on **A** MT1A and **B** MT2 genes were analyzed by ChIP analysis. Genomic DNA extracted from hepatocytes of sham mice and mice with CCl_4_-induced hepatic fibrosis were immunoprecipitated with anti-MTF-1. The alteration in the accumulation of these proteins on metallothionein genes was quantified by qPCR. The data were expressed as a percent of input. An asterisk (*) indicates *p* < 0.05, ** indicates *p* < 0.01, and *** indicates *p* < 0.001. All data are represented as the mean ± SD (*n* = 4)

**Fig. 4:**
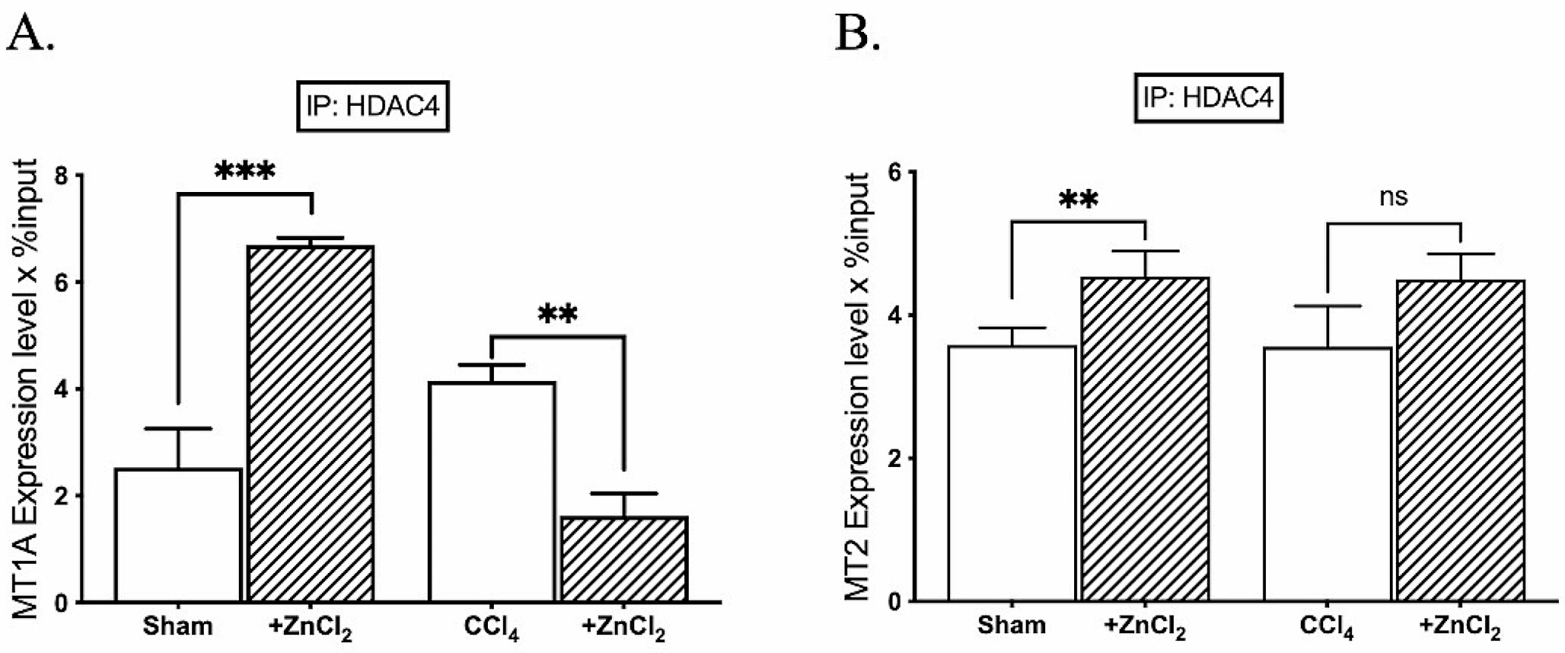
Effects of ZnCl_2_ on the accumulation of histone deacetylase 4 to the promoters of metallothionein genes. The Sham group received mineral oil only, CCl_4_ and CCl_4_ + ZnCl_2_ group were injected with 10% CCl_4_ in mineral oil for 8 weeks. A 10 µM ZnCl_2_ was only injected to CCl_4_ + ZnCl_2_ group for two weeks after CCl_4_ treatment was completed. The enrichment of **A-B** HDAC4 on MT1A and MT2 genes were analyzed by ChIP analysis. Genomic DNA extracted from hepatocytes of sham mice and mice with CCl_4_-induced hepatic fibrosis were immunoprecipitated with anti-HDAC4. The alteration in the accumulation of these proteins on metallothionein genes was quantified by qPCR. The data were expressed as a percent of input. An asterisk (*) indicates *p* < 0.05, ** indicates *p* < 0.01, and *** indicates *p* < 0.001. All data are represented as the mean ± SD (*n* = 4)

Following ZnCl_2_ treatment, we observed a concomitant increase in *MTF-1* expression along with elevated Zn^2+^ levels (Fig. 2A). However, despite this increase in MTF-1 binding, *MT1A* mRNA expression did not proportionally rise; instead, a decline in mRNA expression of both *MT1A* and *MT2* was observed in other groups, coinciding with decreased MTF-1 binding (Fig. 2B). We further investigated MTF-1 binding to the *MT1A* and *MT2* promoters. Intriguingly, the percentage of bound MTF-1 displayed a specific increase solely in the *MT1A* promoter of the sham group (Fig. 3A). Yet, the percentage of MTF-1 binding to *MT2* promote decreased in both sham and fibrosis groups after the ZnCl_2_ treatment (Fig. 3B). Regarding HDAC4 binding to *MT1A* and *MT2* promoters, it showed an increase in MT1A of sham group after ZnCl_2_ treatment amd an anticipated increase in *MT2* across all groups (Fig. 4). However, only the CCl_4_ group exhibited a decrease in the percentage of HDAC4 binding to *MT1A*, contrary to the mRNA expression of *MT1A*. Further exploration of these intricate interactions holds the promise of advancing our comprehension of the epigenetic basis of liver fibrosis and its modulation through zinc supplementation.

**Fig. 5:**
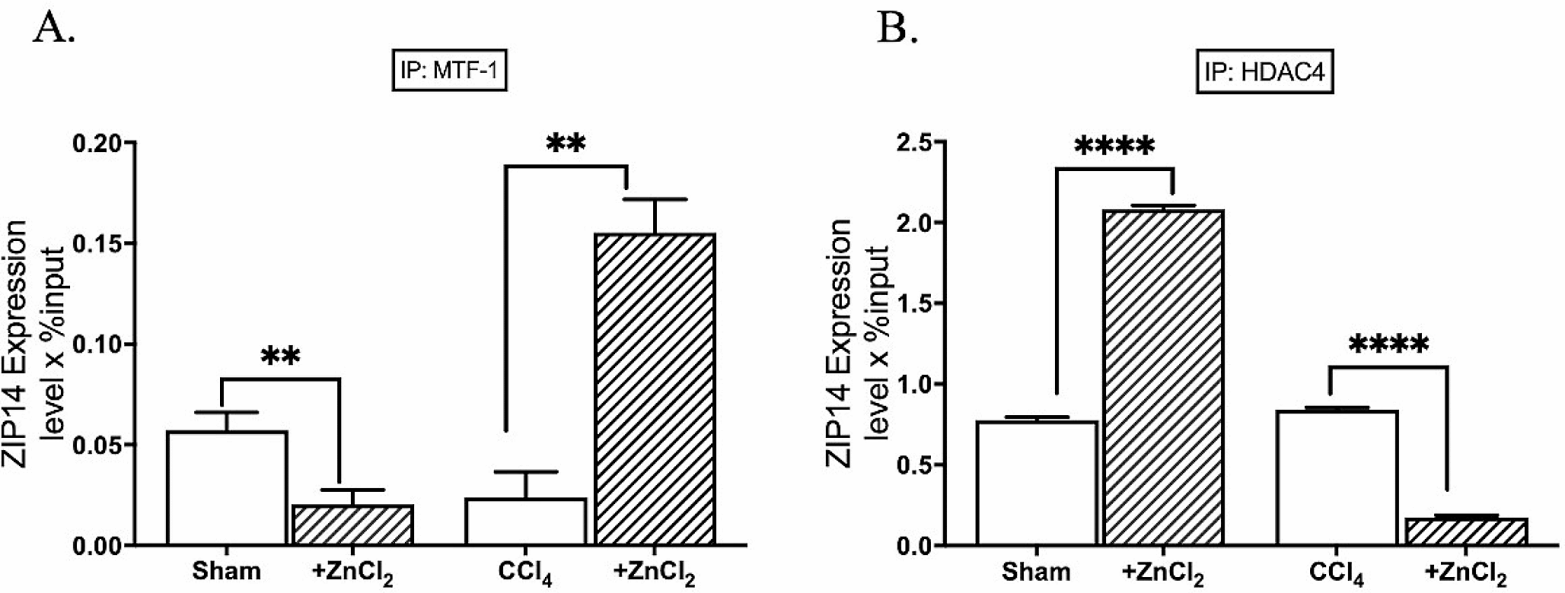
Effects of ZnCl_2_ on the accumulation of MTF-1, histone deacetylase 4 to the promoters of ZIP14 promoter. The Sham group received mineral oil only, CCl_4_ and CCl_4_ + ZnCl_2_ group were injected with 10% CCl_4_ in mineral oil for 8 weeks. 10 µM ZnCl_2_ was only injected to CCl_4_ + ZnCl_2_ group for two weeks after CCl_4_ treatment was completed. The enrichment of **A** MTF-1, **B** HDAC4 on the ZIP14 gene was analyzed by ChIP analysis. Genomic DNA extracted from hepatocytes of **A** control mice and **B** mice with CCl_4_-induced hepatic fibrosis were immunoprecipitated with anti-MTF-1 and anti-HDAC4. The change in the accumulation of these proteins on the ZIP14 gene was quantified by qPCR. The data were expressed as a percent of input. An asterisk (*) indicates *p* < 0.05, ** indicates *p* < 0.01, and *** indicates *p* < 0.001. All data are represented as the mean ± SD (*n* = 4)

**Fig. 6:**
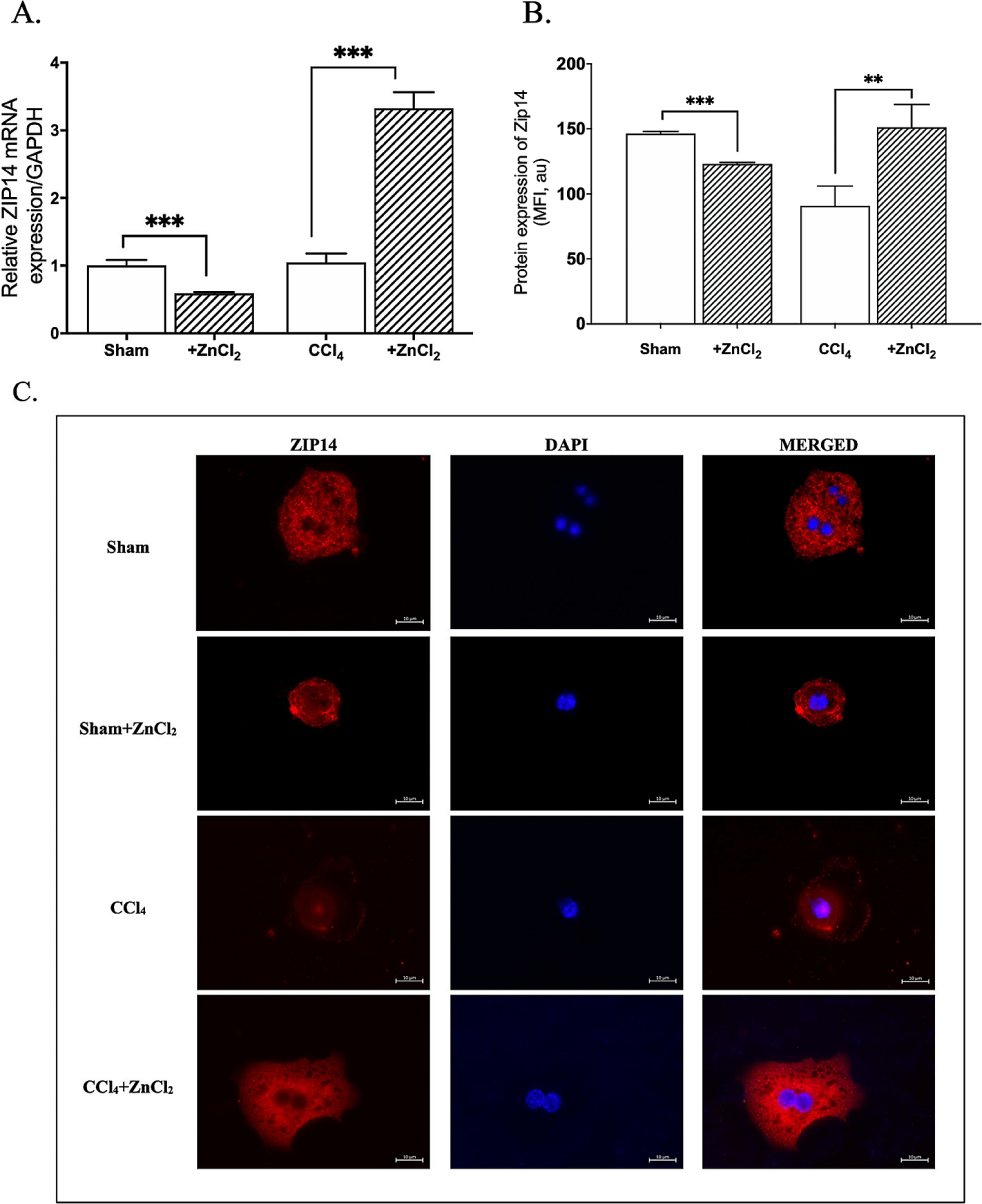
Changes in mRNA and protein expression of the zinc transporter ZIP14 upon ZnCl_2_ treatment in mice with CCl_4_-induced hepatic fibrosis. The Sham group received mineral oil only, CCl_4_ and CCl_4_ + ZnCl_2_ group were injected with 10% CCl_4_ in mineral oil for 8 weeks. A 10 µM ZnCl_2_ was only injected into the CCl_4_ + ZnCl_2_ group for two weeks after CCl_4_ treatment was completed. **A** The mRNA expression levels of ZIP14 in were measured by qRT-PCR in hepatocytes. **B** The protein expression of ZIP14 was measured with flow cytometry and **C** supported with the immunofluorescence staining with ZIP14 antibody. Hepatocytes were isolated from control (sham) and fibrotic (CCl_4_) mice were cultured. Transcript levels were normalized to GAPDH. * Indicates *p* < 0.05, ** indicates *p* < 0.01. All data are represented as the mean ± SD (*n* = 4)

The presence of MRE sequences in ZIP proteins was thoroughly investigated, revealing the presence of the MRE consensus sequence (-2470, TGCACAC) on the promoter of *ZIP14*. Subsequently, we sought to determine whether the transcriptional regulator, MTF-1, binds to the *ZIP14* promoter. Our chromatin immunoprecipitation (ChIP) analyses demonstrated a clear binding of MTF-1 to *ZIP14*, with a noteworthy increase in binding percentage observed in the fibrosis group following ZnCl_2_ treatment, but not in the sham group (Fig. 5A). The transcriptional factor binding increase was accompanied by a significant decrease in the percentage of HDAC4 binding on the *ZIP14* promoter in the hepatocytes of fibrotic group (Fig. 5B). Notably, ZIP14 expression levels exhibited a direct correlation with the bound MTF-1% across all experimental groups. Moreover, subsequent ZnCl_2_ treatment prompted a remarkable elevation of ZIP14 expression, both at the transcript (Fig. 6A) and protein levels (Fig. 6B), in the fibrosis group, while it induced a reduction in expression within the sham group. In the conducted study, immunofluorescent images obtained revealed an intense localization of ZIP14 in fibrotic hepatocytes on the cell membrane. In the hepatocytes of the control group, there was a general distribution; however, following the application of ZnCl_2_ to the control group, specific localizations of ZIP14 expression were observed in the immunofluorescent images. Nevertheless, detailed organelle stainings were not performed within the scope of this study, preventing speculation about the organelles where ZIP14 localization intensified. Additionally, examination of fibrotic hepatocytes treated with ZnCl_2_ showed a significant increase in ZIP14 expression, displaying a distribution similar to that of the control group (Fig. 6C).

Collectively, these findings highlight the complex interplay between MTF-1, epigenetic regulators, and ZIP14 in the context of liver fibrosis and zinc supplementation. The observed dynamic changes in transcription factor binding and acetylation motifs on the *ZIP14* provide critical insights into the regulatory mechanisms underlying the therapeutic effects of ZnCl_2_ treatment on liver fibrosis. Further investigations into these intricate molecular interactions hold significant promise for unraveling the epigenetic basis of liver fibrosis and its modulation through zinc supplementation.

## Discussion

The principal finding of this study is that zinc supplementation in mice with liver fibrosis elicits a promising response, potentially reversing fibrotic characteristics through the modulation of the ZIP14 channel protein, affecting its expression at both genetic and epigenetic levels. The [Zn^2+^]_i_ homeostasis is established at most through zinc transporters located on the cell membrane and the membranes of organelles. In the context of inflammation, an increase in inflammatory factors in the body promotes zinc transfer from plasma to the liver [14]. In our investigation, we confirmed the presence of MRE sequences on the promoter of the ZIP14 gene, and intriguingly, our results demonstrated that MTF-1 regulates ZIP14 when zinc supplementation is administered in the fibrosis groups. This observation suggests that MTF-1 might act as a genetic regulator of zinc’s therapeutic effect on fibrosis. In addition, we also found evidence that ZIP14 expression is subjected to epigenetic regulation in hepatic fibrosis.

Liver fibrosis is characterized by an aberrant wound-healing process that leads to excessive deposition of ECM, particularly fibrillary collagen [2]. While fibrotic tissue formation is a natural tissue healing response, uncontrolled progression can result in severe scar tissue formation and subsequent disruption of intra-tissue blood flow. Prolonged inflammation, necrosis, and abnormal regeneration may culminate in the development of liver fibrosis into cirrhosis and ultimately HCC [33]. The zinc particularly as Zn^2+^, a crucial trace element in cells, plays a pivotal role in various cellular processes as a component of transcription factors and epigenetic modulators. Reduced serum zinc levels are observed in hepatic fibrosis and subsequent HCC. Previous in vivo and in vitro studies have demonstrated the beneficial impact of zinc supplementation on liver fibrosis, particularly in its early stages [4]. However, the precise anti-fibrotic mechanism underlying zinc treatment remains incompletely understood. Hence, exploring the distinct responses of fibrotic and HCC cells to zinc administration could advance our comprehension of the cellular healing mechanisms involved in the therapeutic effects of zinc compounds on liver fibrosis.

In this regard, we examined [Zn^2+^]_i_ concentration in hepatocytes isolated from healthy and fibrotic livers. Remarkably, the [Zn^2+^]_i_ were significantly lower in mice with liver fibrosis, while intraperitoneal injection of 10 µM ZnCl_2_ effectively restored the decreased [Zn^2+^]_i_. The lower [Zn^2+^]_i_ were expected in the liver fibrosis model. Furthermore, we established a robust correlation between [Zn^2+^]_i_ in hepatocytes and the expression levels of αSMA, a well-known fibrosis marker, strongly suggesting that increased [Zn^2+^]_i_ may influence the progression of liver fibrosis. Previous studies by Mohammad et al. and Rodriguez-Moreno have already discussed the association between the [Zn^2+^]_i_ and liver diseases, with a decrease in serum zinc correlating with the progression of liver diseases [4, 42, 43]. Additionally, in another study published in 2023, it was demonstrated that zinc deficiency resulting from pregnancy leads to liver damage in rats [36]. Notably, short-term studies have not reported any adverse reactions to zinc supplementation [4, 37].

Zinc exerts its influence through its high binding affinity to proteins with zinc finger motifs, such as MTs, which play a critical role in regulating [Zn^2+^]_i_. In addition to mitochondria and the ER, MTs act as the primary [Zn^2+^]_i_ reservoirs [8]. In cases where the [Zn^2+^]_i_ falls below the required level, MTs undergo proteolysis, releasing stored Zn^2+^ to restore [Zn^2+^]_i_ homeostasis [38]. MTF-1 serves as a transcriptional regulator, enabling cells to adapt to stress conditions like heavy metal exposure, hypoxia, and oxidative phosphorylation. MTF-1 binds to the MRE sequence as a transcription factor, with its primary function executed through MT proteins [28, 29]. Under physiological conditions, MTF-1 is present in an inactive form in the cytoplasm but is activated in response to cellular stress. Importantly, while MTF-1 can be activated by other heavy metals, it requires sufficient Zn^2+^ for binding to the MRE sequence [26]. In our investigation, we aimed to comprehend [Zn^2+^]_i_ homeostasis and the role of MTs in hepatic fibrosis. Interestingly, elevated [Zn^2+^]_i_ increased MTF-1 expression levels but did not significantly affect MTF-1 binding percentage on MT1A and MT2 genes, both in the control and fibrotic groups. Despite the increased MTF-1 binding percentage on the MT1A promoter in control mice, there was no correlated increase in MT1A mRNA levels. Also, MTF-1 binding on MT1A and MT2 promoters, as well as mRNA levels of both MTs, were decreased in our findings. This led us to speculate that epigenetic regulation might take precedence over the cooperation of MTF-1 and MTs in the cellular regulation of [Zn^2+^]_i_ under fibrotic conditions.

The HDACs, except class III, contain a zinc-binding domain and necessitate Zn^2+^ for functional deacetylase activity. Serum zinc deficiency caused by liver fibrosis leads to a decrease in HDAC activity [39]. HDACs play important roles in many physiological and pathophysiological processes. Previous studies have shown the significant involvement of class IIa HDACs in HSC activation, particularly in fibrosis formation. A previous study has demonstrated the crucial impact of HDAC4 in regulating ECM deposition in a liver fibrosis model induced by CCL_4_ [40]. To investigate the effect of HDAC4 on hepatic fibrosis and to examine its role not only in ECM deposition but also in different regulations following zinc treatment, a ChIP assay was conducted with HDAC4. Our examination of HDAC4 binding on MTs revealed a significant increase in HDAC binding on the MT1A promoter in the control group, possibly explaining the decreased MT1A expression despite the increased MTF-1 binding percentage on the same promoter. The decrease in MT2 expression could be attributed to both an increasing percentage of HDAC4 binding and a decrease in MTF-1 binding on its promoter. However, in the fibrosis group, MT1A expression decreased following zinc supplementation despite the increased [Zn^2+^]_i_ and reduced HDAC4 binding percentage. These findings led us to consider that other factors might orchestrate the regulatory process under fibrotic conditions. While MT proteins are well-known as the primary intracellular zinc storages, we postulated that the stabilization of [Zn^2+^]_i_ homeostasis might be governed not only through the MTF-1 and MT1A-MT2 axis but also through the regulation by other factors such as zinc transporters.

Overall, considering our present findings together with literature data, zinc supplementation appears to elicit a positive response at the cellular level through epigenetic rearrangements. Our results showed that zinc supplementation increases the percentage of MTF-1 binding on the ZIP14 promoter in liver fibrosis. This increase in MTF-1 binding is consistent with the observed elevation in ZIP14 expression levels. Furthermore, we demonstrated that HDAC4 activity on the ZIP14 promoter was reduced, providing supporting evidence, along with the abundance of H4K5ac and H4K12ac motifs on the ZIP14 promoter. Our study indicates that CCl_4_-induced hepatic fibrosis leads to decreased intracellular zinc concentrations, and zinc accumulation in hepatocytes might be mediated by ZIP14 following zinc supplementation in mice with liver fibrosis. ZIP14 is extensively studied as a zinc transporter in liver diseases. Studies by Kim et al. have shown that ZIP14-/- knockout mice exhibit impaired hepatic zinc uptake and suggested that ZIP14 plays a crucial role in cellular adaptation to ER stress, which in turn plays a distinct role in hepatic fibrosis [15]. Another study has demonstrated that physiological stress also induces ZIP14 expression in the liver as part of the cellular response to stress conditions [16]. To the best of our knowledge, this study provides novel insights into the regulation of [Zn^2+^]_i_ homeostasis in hepatic fibrosis.

In the context of inflammation, an increase in inflammatory factors in the body promotes Zn^2+^ transfer from plasma to the liver [41]. In our investigation, we confirmed the presence of MRE sequences on the promoter of the ZIP14 gene, and intriguingly, our results demonstrated that MTF-1 regulates ZIP14 when zinc supplementation is administered in the control groups. This observation suggests that MTF-1 might act as a genetic regulator of zinc’s therapeutic effect on fibrosis. However, we also found evidence that ZIP14 expression is subjected to epigenetic regulation in hepatic fibrosis.

Therefore, zinc supplementation appears to elicit a positive response at the cellular level through epigenetic rearrangements. Our results showed that zinc supplementation increases the percentage of MTF-1 binding on the ZIP14 promoter in liver fibrosis. This increase in MTF-1 binding is consistent with the observed elevation in ZIP14 expression levels. Furthermore, we demonstrated that HDAC4 activity on the ZIP14 promoter was reduced, providing supporting evidence. Our study indicates that CCl_4_-induced hepatic fibrosis leads to decreased intracellular zinc concentrations, and zinc accumulation might be mediated by ZIP14 following zinc supplementation in mice with liver fibrosis. ZIP14 is extensively studied as a zinc transporter in liver diseases. Studies by Kim et al. have shown that ZIP14^-/-^ knockout mice exhibit impaired hepatic Zn^2+^ uptake and suggested that ZIP14 plays a crucial role in cellular adaptation to ER stress, which in turn plays a distinct role in hepatic fibrosis [42]. Another study has demonstrated that physiological stress also induces ZIP14 expression in the liver as part of the cellular response to stress conditions [43]. To the best of our knowledge, this study provides novel insights into the regulation of [Zn^2+^]_i_ homeostasis in hepatic fibrosis.

## Conclusions

The deficiency of serum zinc levels is a common characteristic observed in nearly all types of liver diseases. In our study, we demonstrated that liver fibrosis profoundly influences [Zn^2+^]_i_ homeostasis, and importantly, we found that [Zn^2+^]_i_ can be effectively restored even under conditions of hepatic fibrosis. Previous investigations have attributed the regulation of [Zn^2+^]_i_ homeostasis primarily to ZIP/ZnT and MT proteins under physiological circumstances. However, our findings propose that the MTF- MT1A-MT2 axis may not solely account for the increased free zinc levels observed in hepatic fibrosis after ZnCl_2_ administration, but rather, zinc transporter channels may also play a direct role in the regulation of [Zn^2+^]_i_ homeostasis in this context. Significantly, we have provided novel evidence, for the first time in the literature, elucidating that MTF-1 exerts control over ZIP14 expression in conjunction with histone deacetylase activity following zinc supplementation in hepatocytes afflicted with hepatic fibrosis. Our results suggest that ZIP14 may serve as a primary factor responsible for the maintenance and regulation of [Zn^2+^]_i_ homeostasis in liver fibrosis.
